# A Flexible, Implantable, Bioelectronic Electroporation Device for Targeted Ablation of Seizure Foci in the Mouse Brain

**DOI:** 10.3390/s25010004

**Published:** 2024-12-24

**Authors:** Rita Matta, Zsofia Balogh-Lantos, Zoltan Fekete, Martin Baca, Attila Kaszas, David Moreau, Rodney Philip O’Connor

**Affiliations:** 1 Mines Saint-Etienne, Centre CMP, Département BEL, F-13541 Gardanne, France; rita.matta@emse.fr (R.M.); martin.baca@emse.fr (M.B.); 2 Research Group for Implantable Microsystems, Faculty of Information Technology & Bionics, Pazmany Peter Catholic University, H-1083 Budapest, Hungary; lantos.zsofia@itk.ppke.hu (Z.B.-L.); fekete.zoltan@itk.ppke.hu (Z.F.); 3 Roska Tamás Doctoral School of Sciences and Technology, Faculty of Information Technology & Bionics, Pazmany Peter Catholic University, H-1083 Budapest, Hungary; 4 Sleep Oscillation Research Group, Institute of Cognitive Neuroscience and Psychology, HUN-REN Research Center for Natural Sciences, Hungarian Research Network, H-1117 Budapest, Hungary; 5 Multimodal Neurotechnology Group, Institute of Cognitive Neuroscience and Psychology, HUN-REN Research Centre for Natural Sciences, Hungarian Research Network, H-1117 Budapest, Hungary

**Keywords:** electroporation, epilepsy, organic electrodes, in vivo, mouse, neuron, two-photon imaging, calcium imaging, microfabrication, flexible electrodes

## Abstract

The primary method of treatment for patients suffering from drug-resistant focal-onset epilepsy is resective surgery, which adversely impacts neurocognitive function. Radio frequency (RF) ablation and laser ablation are the methods with the most promise, achieving seizure-free rates similar to resection but with less negative impact on neurocognitive function. However, there remains a number of concerns and open technical questions about these two methods of thermal ablation, with the primary ones: (1) heating; (2) hemorrhage and bleeding; and (3) poor directionality. Irreversible electroporation (IRE) is a proven method of focal ablation, which circumvents all three of the primary concerns regarding focal RF and laser ablation. Here, we demonstrate the in vivo application of a flexible implant with organic electrodes for focal ablation of epilepsy foci using high-frequency IRE (H-FIRE) in mice. Our results show that local, targeted ablation is possible in the close neighborhood of the electrode, paving the way for the clinical application in the treatment of focal epilepsy.

## 1. Introduction

Epilepsy is a disorder of the brain characterized by an enduring predisposition to generate epileptic seizures [1] that affects around 70 million people worldwide [2]. The appearing recurrent unprovoked seizures can start from a clearly defined area in case of focal seizures or cover multiple brain areas in generalized seizures. The basis of developing seizures lies in the breaking of the excitation−inhibition balance [3,4], allowing for drug-based therapies using anti-seizure drugs (ASDs). However, one-third of the patients suffer from drug-resistant epilepsy [5] and need other therapeutical methods. Though resective surgery of the epileptic focus can provide relief from epileptic seizures, it can impact neurocognitive function. By definition, epileptic seizures are transient spasms of increased neuronal discharge, which results in an event that is discernible to the patient [6]. Seizures can be divided into three categories: generalized onset, focal onset (formerly called partial), and unknown onset [7]. While generalized seizures are produced by electrical impulses from throughout the entire brain [8], focal onset seizures originate in one zone and can extend across the brain, causing moderate to severe symptoms, depending on how the electrical discharges propagate. These focal seizures originate in neuronal networks limited to part of one cerebral hemisphere [9].

Depending on the nature of the seizures, the doctor may propose diet treatment or nerve stimulation [10], but usually, medications (for example, Lamotrigine [11], Levetiracetam [12], and valproic acid [13]) can help most people with epilepsy have fewer seizures or ultimately stop experiencing them. In the case of drug-resistant focal-onset epilepsy, resective surgery would be the primary method of treatment. This implies the removal of a tiny piece of the brain, usually brain tissues from the location of seizures, which is the site of a tumor, brain injury, or malformation [7]. Similar to other brain surgeries, and since it is often conducted on one of the temporal lobes, complications from brain resections may include increased risk of infection, speech or memory problems, stroke, loss of movement or visual abilities, loss of eyesight, or seizures [14]. A significantly less invasive surgical method is laser interstitial thermal therapy (LITT), also known as laser ablation surgery, which can be beneficial for treating drug-resistant focal epilepsy [15,16,17,18]. Here, magnetic resonance imaging (MRI) is performed during surgery to accurately map out the specific area of the brain to operate on [19]. The seizure focus is then removed by the precise delivery of laser to this region [20]. Another minimally invasive treatment is radiofrequency ablation (RFA), also known as radiofrequency neurotomy, which has emerged as a promising therapy for extra-cranial malignancies [21]. The heat kills a section of the nerve tissue, preventing it from transmitting pain signals to the brain. However, there are some consequences of these two ablation techniques: tissue damage due to heating, intracerebral hemorrhage, low accuracy in targeting the seizure focus, and less predictability of lesion size and shape [22].

Electroporation, also known as electropermeabilization, is a biophysical phenomenon, inducing the permeabilization of the cell membranes, occurring with the application of short and intense electric fields across cells or tissues [23,24]. Depending on the physical parameters used, i.e., electric field amplitude and duration, number of pulses and frequency, the permeabilization may be either temporary (reversible electroporation) or permanent (irreversible electroporation, IRE). While reversible electroporation is commonly used in vitro to facilitate the penetration of various otherwise non-permeable macromolecules across the cell membrane, IRE is a non-thermal ablation technique [25] allowing one to induce permanent nanoscale defects on membranes, resulting in the disruption of cell homeostasis and leading to their death [26,27].

The irreversible electroporation ablation technique uses a train of high-amplitude microsecond electrical pulses [28,29]. High-frequency IRE (H-FIRE) has benefits over other ablative technologies in terms of treatment speed (minutes) [30], and the possibility for sparing of the vasculature, ductal networks, and extracellular matrix, which aids post-therapy recovery [31]. When compared to other treatments, it has a reduced risk of hemorrhage [30,32,33] and allows improved precision in brain applications [30,34,35,36]. Though H-FIRE has been used in the studies for the treatment of prostate cancer [37,38] or studies on hepatic ablation [39], lung cancer [40], or gliomas [27,35], its role in the treatment of focal-onset epilepsy has been poorly considered [33].

Furthermore, inserting rigid electrode materials into the brain induces trauma into the tissue, due to mechanical mismatches and foreign body inflammatory responses [41,42]. This aspect might be solved with the emergence of plastic bioelectronic technologies. Indeed, thin-film electrodes have a lower Young’s modulus compared to metal- and silicon-based electrodes [43], and their flexibility allows a better compliance with biological tissues [44].

Here, we propose a flexible implant with organic electrodes for focal ablation of epilepsy foci using irreversible electroporation. By using flexible organic microelectrode arrays (MEAs), we endeavor to minimize the damage caused by the penetration of the electrodes in the brain tissue and that cause all adverse reactions associated therein. After the description of their fabrication, we show the effect of IRE protocols applied using thin-film electrodes when implanted onto the surface of the brain of GCaMP6f mice. We characterize the induced calcium signals and their spatial selectivity. Finally, we show the effect of IRE on a chemical model of epilepsy.

## 2. Materials and Methods

### 2.1. MEA Fabrication

Briefly, 2 µm Parylene C was deposited on a clean glass slide through a regulated chemical vapor deposition process (SCS Labcoater 2, Speciality Coating Systems Inc., Indianapolis, IN, USA). Gold electrodes and connection leads were patterned through the use of a lift-off process. A bi-layer of LOR5A resist (Kayaku Advanced Materials, Westborough, MA, USA) and S1813 photoresist (Kayaku Advanced Materials, Westborough, MA, USA) spin-coated at 3000 rpm for 35 s and 3500 rpm for 35 s, respectively, was exposed with a SUSS MBJ4 (SUSS MicroTec, Munich, Germany) contact Mask aligner through a photomask (Selba SA, Versoix, Switzerland) containing the design (h-line, λ = 405 nm, energy density = 130 mJ·cm^−2^). Then, thermal evaporation was used to deposit an adhesion layer of 10 nm chromium followed by a layer of 150 nm of gold (metals provided by Neyco, Vanves, France) using a Boc Edwards thermal evaporator (Edwards, Burgress Hill, UK). After lift-off, another 2 µm Parylene C insulation layer was deposited with 3-(trimethoxysilyl)propyl methacrylate (A-174 Silane) as an adhesion promotor. After another step of photolithography with AZ9260 photoresist spin-coated at 3000 rpm for 35 s and exposed at λ = 405 nm, energy density = 280 mJ·cm^−2^ (Microchemicals GmbH, Ulm, Germany), reactive ion etching (CF_4_: 10 sccm, O_2_: 50 sccm, RF power: 200 W; Plasmalab80Plus, Oxford Instruments, Abingdon-on-Thames, UK) was used to etch the outline of the probe. Final steps intended to coat the electrodes with PEDOT:PSS. To do so, a soap layer (2% in water) was spin-coated before the deposition of a 2 µm sacrificial layer of Parylene C. AZ9260 was used again for the photolithography before reactive ion etching of both sacrificial and insulation layers of Parylene C on top of the e in-between at 110 °C on a hotplate. The sacrificial Parylene C layer was peeled-off followed by a 1 h bake at 140 °C. Finally, the device was immersed in deionized water to remove excess low-molecular-weight compounds and to aid in the release of telectrodes. A mixture of PEDOT:PSS (Heraeus Clevios PH1000, Heraeus, Hanau, Germany), ethylene glycol, dodecyl benzene sulfonic acid, and (3-glycidyloxypropyl) trimethoxysilane was spin-coated twice (3000 and 1500 rpm) with a one-minute bakhe device from the glass slide. The device was then attached to a ZIF cable (Digikey, Thief River Falls, MN, USA), with an anisotropic conductive film (Digikey, Thief River Falls, MN, USA) as a connecting layer between the probe and the ZIF cable, secured afterwards with Kapton tape and epoxy resin to make it impermeable and resistant to light and heat.

### 2.2. Electrochemical Characterization

**Figure 1:**
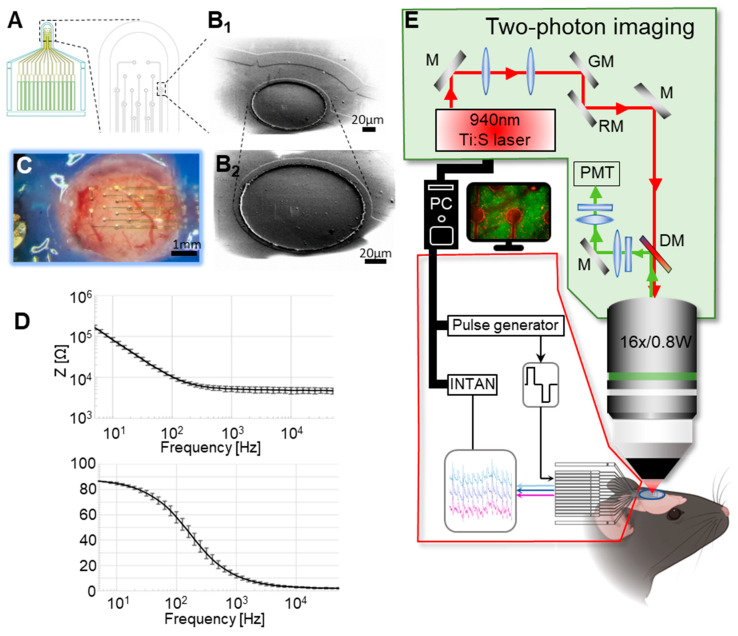
Device characteristics and two-photon imaging setup. (**A**) Schematic drawing of the device layout. (**B1**,**B2**) Scanning electron micrograph of one electrode pad (**B1**) with an adjacent connecting line (**B2**) at high magnification. (**C**) The implanted device above the mouse visual cortex. (**D**) Representative electrochemical impedance spectroscopy (EIS) characterization of one 14-channel device (representations as mean ± STD). (**E**) Imaging and stimulation scheme.

Electrochemical characterization was performed through the PalmSens 4 portable potentiostat (PalmSens BV, Utrecht, The Netherlands). Electrochemical impedance spectroscopy was performed in a two-electrode setup where the working electrode was one electrode of the MEA and the reference and counter electrodes were both connected to an Ag/AgCl electrode immersed in PBS. Measurements of impedances were made through the use of a 10 mV sinusoidal voltage with a frequency varying from 5 to 50,000 Hz (Figure 1D).

### 2.3. Animal Experimentation

Adult Thy1-GCaMP6f mice (*n* = 3 males; 25–72 weeks old) were housed according to French regulations in the animal facility of Institute de Neurosciences de la Timone (Authorization number: 22689-2019100414103054). The surgical procedures was conducted as described previously [45]. Briefly, on the day of surgery, animals were sedated with 3% sevoflurane and fixed in a stereotactic frame. For the H-FIRE protocol, we prepared a 4 cm long copper wire that had a single-pin jumper connector soldered at one end and a medical-grade self-tapping bone screw (FST, Heidelberg, Germany) at the other. After a subcutaneous lidocaine injection, the skull was exposed, and the screw was implanted according to the mouse brain stereotactic atlas [46] at the following coordinates: anteroposterior (AP) 2 mm and mediolateral (ML) 1.7 mm. Similarly, a cranial window (4 mm diameter) was opened above the visual cortex (centered at AP −2.5 mm and ML 2.2 mm). The electrodes were placed directly on the dura and covered with a custom glass coverslip that was cut to shape to accommodate for an injection pipette at the side of the device and the craniotomy. A custom-made stainless steel head plate was mounted onto the skull by light-curing dental resin, and the animal was placed into the microscope setup using the head plate. To visualize electroporation, the astrocyte-specific dye Sulforhodamine-101 (150 µM) [47] with 25 mM 4-aminopyridine (4-AP) for inducing seizure-like activity [48,49] was injected under two-photon visual guidance beneath the selected electrode pads. During the recording procedure, the anesthesia was maintained with a 0.9% isoflurane and oxygen mixture.

### 2.4. In Vivo Two-Photon Calcium Imaging

After a brief induced sedation by 2% isoflurane, the animal temperature was maintained at 38 °C by its placement on a heating pad. Its head was fixed by screwing the head-fixed metal bar to the custom mouse holder. Anesthesia was maintained with a 0.9% isoflurane and carbogen mixture during the imaging procedure. An ultrasound transmission gel (OptiLube, EcoMed Services, Antwerp, Belgium) was applied on the top of the optical window as an immersion medium for the objective used (Nikon CFI75 LWD 16 × W). Two-photon images were acquired on a dual-scanning-head two-photon microscope (FemtoS-Dual, Femtonics Ltd., Budapest, Hungary) equipped with a femtosecond-pulsed laser tuned to 920 nm (Mai Tai HP, SpectraPhysics, Mountain View, CA, USA). A single acquisition plane was selected, and full-frame imaging was started in resonant scanning mode at 30.9375 Hz. Laser intensity was set between 5 mW and 120 mW for 0 to 600 µm in depth, respectively, for z-stack recordings. Time sequences were acquired with 35 mW at 250 µm under the surface. The control of calcium signal recording and the trigger of H-FIRE protocols were performed using the microscope’s acquisition software (MESc v4.6, Femtonics Ltd., Budapest, Hungary).

### 2.5. H-FIRE Protocol

**Figure 2:**
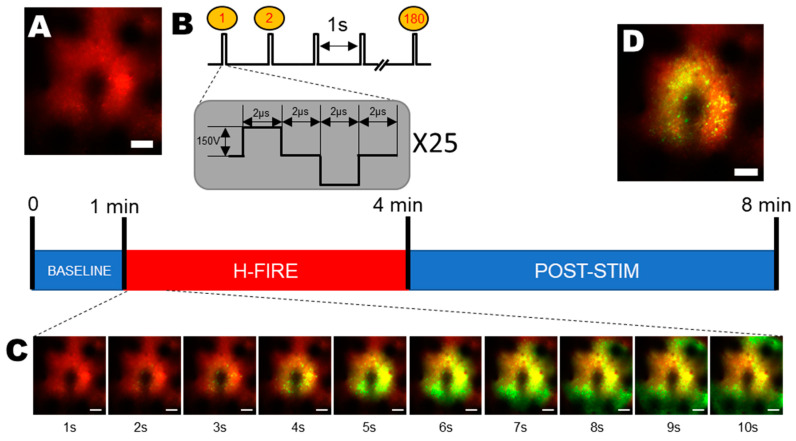
Experimental timeline. The recordings started with a 1 min-long baseline period (**A**), and then, the H-FIRE protocol was triggered (**B**). (**C**) The evoked calcium waves right after the H-FIRE stimulation. (**D**) The post-stimulation lasting for 4 min. Images were averaged over 1 s intervals.

Electrical pulses were delivered using a custom-made pulse generator. We used the IRE protocol described previously [26]. Briefly, our protocol was delivered 1 h and 30 min after the injection of 4AP using a 250 kHz delayed protocol (Figure 2B) consisting of 25 bipolar pulses (2 µs positive polarity, 2 µs break, and 2 µs negative polarity) on the selected stimulation site of our probe, with the implanted surgical screw 4 mm away from the stimulation site serving as the ground. The pulse train was run 180 times with a repetition frequency of 1 Hz. The voltage (150 V) was set with a precision of 1 V at the desired value.

### 2.6. Electrophysiological Recordings

The device electrode pads were used not just for the stimulation, but also for multichannel local field potential (LFP) recordings. The devices were connected to a multichannel headstage (RHS2116, INTAN Technologies, Los Angeles, CA, USA) through a ZIF-CLIP connector (Omnetics, Minneapolis, MN, USA), while the headstage was connected through a data cable (Stim SPI cable, INTAN Technologies, Los Angeles, CA, USA) to a 128-channel recording/stimulation system (RHS stim/recording controller, INTAN Technologies, Los Angeles, CA, USA). Recordings were either started separately from imaging or were triggered by the start of the imaging session using the MESc microscope software (Femtonics, Budapest, Hungary). Data acquisition, display, and analysis were performed using the INTAN RHX Data Acquisition Software v 3.0.6 (INTAN Technologies, Los Angeles, CA, USA).

### 2.7. Segmentation of Neurons in Two-Photon Images

The segmentation of the neurons was conducted automatically using a custom MATLAB script. First, the last 20 s of the two-photon measurement was loaded and averaged to get the image for the segmentation. After that, the image was padded and cut into overlapping, 238-by-238 tiles. The segmentation was performed on these individually. At the beginning of the segmentation, a Wiener filter with a 3-by-3 neighborhood and a Gauss (imgaussfilt) filter with a standard deviation of 0.5 were used to remove the noise [50]. The Wiener filter uses a pixel-wise adaptive method based on the local mean and variance around each pixel:

(1) $$\mu =\frac{1}{NM}\sum_{n_{1},n_{2}\epsilon \eta }a(n_{1},n_{2})$$

(2) $$\sigma^{2}=\frac{1}{NM}\sum_{n_{1},n_{2}\epsilon \eta }a^{2}\left(n_{1},n_{2}\right)-\mu^{2}$$

where η is the N-by-M neighborhood of each pixel.

Then, the function creates a pixel-wise Wiener filter:

(3) $$b\left(n_{1},n_{2}\right)=\mu +\frac{\sigma^{2}-\nu^{2}}{\sigma^{2}}(a\left(n_{1},n_{2}\right)-\mu )$$

where $\nu^{2}$ is the noise variance.

The Gauss filter is a smoothing linear filter which uses the Gaussian function:

(4) $$G\left(x,y\right)=\frac{1}{\sqrt{2\pi \sigma^{2}}}e^{-\frac{x^{2}+y^{2}}{2\sigma^{2}}}$$

where σ is the standard deviation of the distribution, which is assumed to have a mean of 0.

To enhance the contrast of the image, contrast-limited adaptive histogram equalization (adapthisteq) was used [51]. This method computes several histograms and uses them to redistribute the intensity values. The noise overamplification is avoided by the limitation of the amplification.

The objects were eliminated on the borders (imclearborder), and the tiles were binarized (imbinarize) based on the average intensity to extract the perimeters of the cells. If the mean intensity value of the tile was greater than 0.1, an adaptive threshold was used with a sensitivity value of 0.006. This threshold was chosen using local first-order image statistics around each pixel. If the mean intensity was less than 0.1, a global threshold was used based on Otsu’s method [52]. This method chooses a threshold that minimizes the variance of the thresholded black and white pixels. This way, the segmentation can be more accurate on tiles with a different number of cells. Morphological operations (imfill strel, imopen) were also used to structure the images, which makes the objects more visible.

For accurate segmentation, it is necessary to separate the cells from each other. For this, the watershed algorithm was used [53]. This algorithm treats the images as surfaces, where light pixels are elevations and dark pixels represent wells. The sure background pixels are determined using morphological operations, and the sure foreground pixels are found by calculating the distance transform. The unknown areas are the markers for the algorithm to determine the exact boundaries. Later on, each detected object was labeled and filtered based on circularities with a threshold value of 0.4, so the neurites and other noises were filtered out.

After the segmentation, the coordinates of the detected cell bodies were mapped to the original image, and the double centroids were filtered out. Based on the segmented image, the average intensity change was extracted for every cell body.

### 2.8. Data Analysis

Primarily, two-photon calcium imaging time series were analyzed with the MESc data acquisition software (Femtonics Ltd., Budapest, Hungary) and the MES curve analyzer tool. Manual selection of regions of interest was performed to coincide with the somata of single cells. The raw fluorescence calcium traces were extracted and analyzed using custom Matlab scripts.

The intensity changes were filtered with a 3-point Gaussian window using convolution, followed by the calculation of the relative intensity change (*dF*/*F*):

(5) $$dF=\frac{F_{t}-F_{0}}{F_{0}}$$

where $F_{t}$ is the signal and $F_{0}$ is the baseline. For the baseline, the average of the last 9.5 s before stimulation was used for each intensity change.

Events were detected if the relative intensity change was bigger than a threshold calculated for each cell. The thresholds were determined as standard_deviation_of_the_baseline*15.

For the Plateau detection, abrupt changes in the signal were determined using the changes in root-mean-square (RMS) level (findchangepts) [54,55]. The changepoint is the time when this statistical property of the signal ($x_{1},x_{2},{\ldots ,x}_{N}$) changes abruptly. In the case of the RMS, the total deviation was the same as for standard deviation, but the mean was set to zero:

(6) $$\sum_{i=m}^{n}∆\left(x_{i};\chi \left(\left[x_{m}\ldots x_{n}\right]\right)\right)=(n-m+1)log\left(\frac{1}{n-m+1}\sum_{r=m}^{n}x_{r}^{2}\right)$$

The signal parts between these points were considered as possible plateaus. The interval was a plateau if the interval was longer than 1500 frames (around 52 s) and the mean amplitude of the interval was larger than one-third of the maximum amplitude of the signal.

## 3. Results

The two main objectives of this work were to investigate whether the irreversible electroporation ablation technique was achievable through the use of surface-mounted flexible MEAs and if the focality of the technique was controlled with the use of those devices. To do so, intracellular calcium signals in Layer 2/3 neurons in mouse cortex in vivo were investigated through the acquisition of two-photon calcium fluorescence images from Thy1-GCaMP6f transgenic mouse cortices. In order to obtain a fluorescence baseline, we applied the pulsed electric fields 60 s after the beginning of the recordings. The ablation protocol consisted of 25 bipolar pulses (see Section 2) repeated at 1 Hz (Figure 2B) [26].

**Figure 3:**
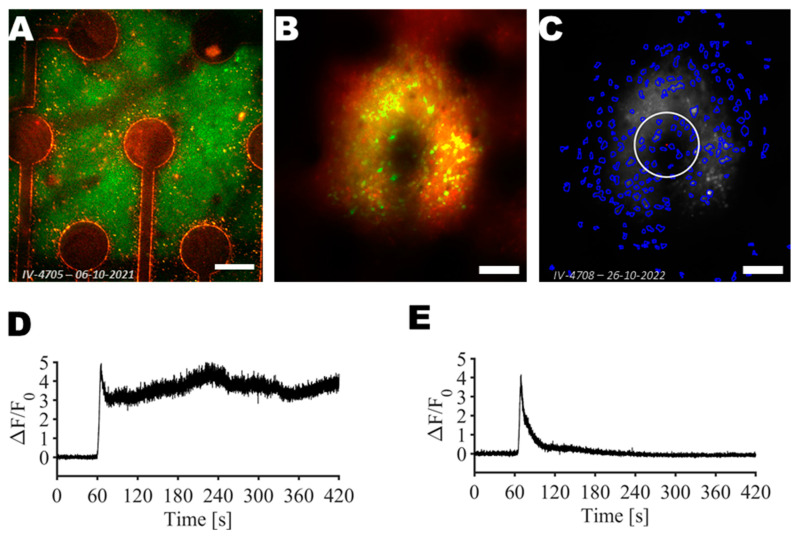
Two-photon imaging and identification of neurons. (**A**) Two-photon image of the probe stimulation/recording sites and connectors. (**B**) Average 7 min after H-FIRE induction and 4 min after H-FIRE protocol was completed. (**C**) Segmented cells outlined in blue based on the last 30 s of the post-stimulation recording (from 3′30″ till 4′ of post-stimulation). White circle shows the electrode area. (**D**) Example of intracellular calcium ion concentration increase in the vicinity of the active electrode (distance from the electrode center = 152 µm). (**E**) Example of intracellular calcium ion concentration increase further from the active electrode (distance from the electrode center = 294 µm). Scale bar: 100 µm.

Following the application of the pulsed electric fields on the central electrode seen in Figure 3, two different evoked intravital calcium signals were observed (see Figure 3D,E and Movie S1). First, in the vicinity of the active electrode, a strong and fast increase of neuronal intracellular calcium was observed. Furthermore, the calcium concentration in the subset of neurons remained elevated. Further from the electrodes, neuronal intracellular calcium signals showed delayed and reversible increases.

**Figure 4:**
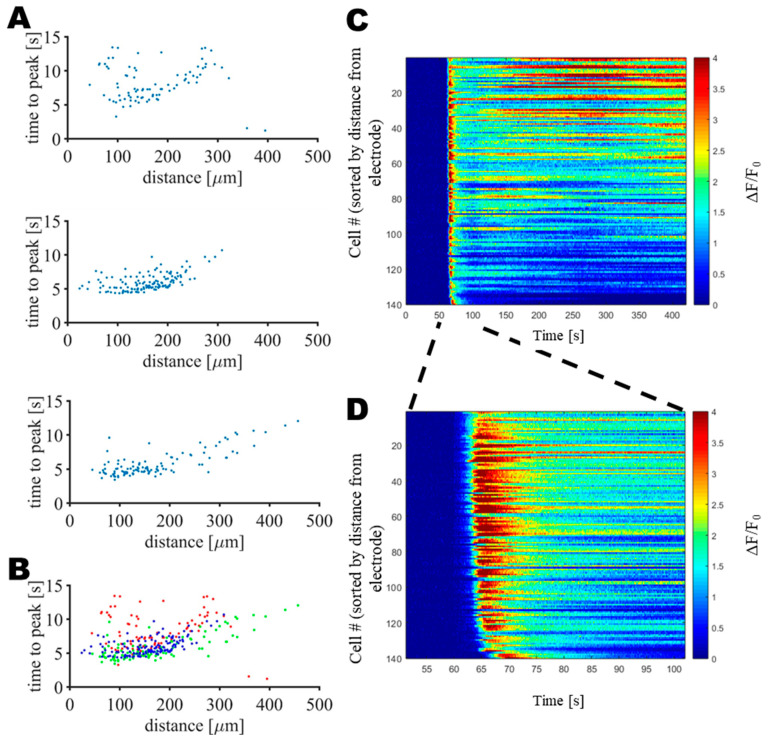
Activation of cells by H-FIRE shows distinct distance dependence. (**A**) Distance dependency of the onset time to the peak of the increase of the intracellular calcium ion concentration for each mouse dataset. (**B**) The overlay of all recorded data from the three animals shown in (**A**). (**C**) Calcium responses as sorted by distance of responding cells from the electrode. (**D**) Neuronal responses when we zoomed in on the time window around the start of the H-FIRE stimulation.

In order to better see the distance dependency of these two subpopulations, neurons were sorted according to their distance from the active electrode and the onset time of the increase in peak intracellular calcium ion concentration was measured, as shown in Figure 4, where each dot represents a single neuron.

Figure 4 shows that in the vicinity of the electrodes (in a radius of 200 µm), the calcium ion concentration was at its maximum 5.9 ± 1.8 s (mean ± SD; N = 246 neurons from 3 mice) after the onset of the pulsed electric fields. Beyond 200 µm, the calcium peak occurred at a time which increased linearly with the distance, in agreement with the calcium wave which can be seen in Movie S1. Altogether, those observations in the calcium signals suggested that within a distance of 200 µm from the electrode, the applied pulsed electric fields induced damage to the tissue through irreversible electroporation as the intracellular calcium ion concentration instantaneously and simultaneously increased and remained high over time. Beyond a distance of 200 µm, a transient and delayed calcium wave was observed. Here, either the H-FIRE stimulation strength decreased gradually beyond 200 µm that it evoked reversible, rather than irreversible, electroporation [56], or the damaged part in the focal area affected glial cells, resulting in a neuronal calcium wave outside the ablation site [57,58].

**Figure 5:**
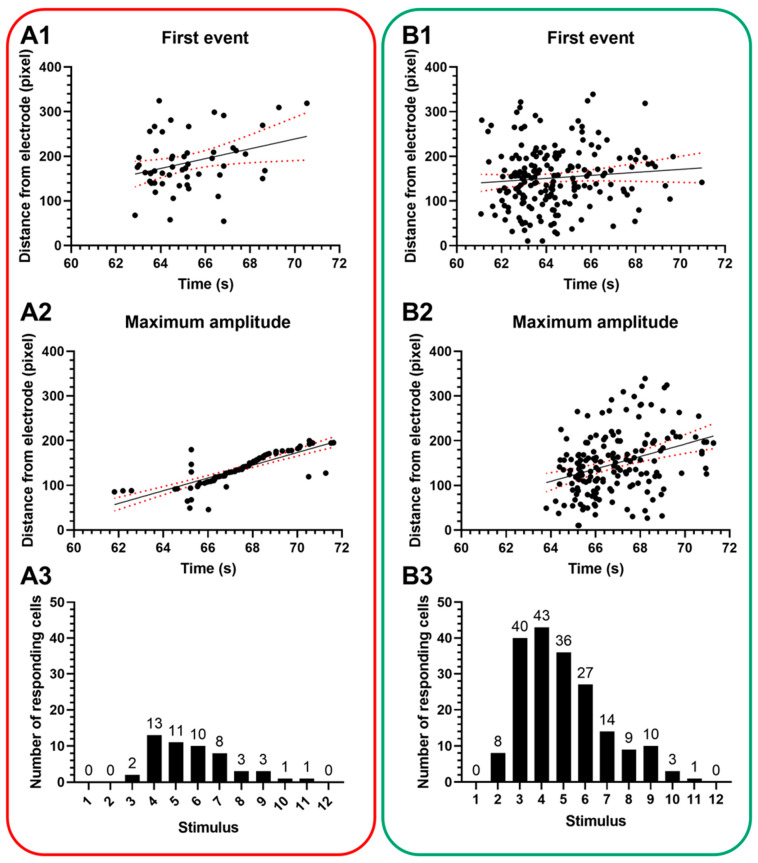
Response distribution of automatically identified neurons after H-FIRE. Distance dependence of H-FIRE evoked neuronal responses for the red (**A1**–**A3**) and the green (**B1**–**B3**) channels for the first evoked events (**A1**,**B1**) and for the maximal response (**A2**,**B2**) of a given neuron. The third row shows the distribution of responding neurons with the number of stimulus bursts applied for the red (**A3**) and the green (**B3**) channels. Red dotted lines show confidence bounds of the quadratic linear regression.

When looking at not just the green channel, but the red channel response distribution (Figure 5) in two-photon imaging, a very similar tendency can be observed. On the other hand, the functionally inactive channel also showed increased fluorescence, indicating that more cells took up greater amounts of the SR-101. This suggested that these cells were more permeable to this dye in the interstitial fluid. Looking at the sequence of stimuli, the response was not immediate (Figure 5A3,B3): the first stimulus did not evoke any response, and it was only from the second and third stimulus trains that we can clearly see cells with increased fluorescence intensity. It was the fourth stimulus train that clearly evoked a response in the largest number of cells, and after that, the number of newly stimulated neurons slowly decreased. The immediate effect on the cells seemed to disappear after 12 s, leading to the conclusion that all cells that could have already reacted, suggesting that any further H-FIRE train would have effects beyond immediate neuronal stimulation.

In order to confirm the local nature of the H-FIRE protocol’s sustained effect, the intracellular fluorescence of GCaMP6f was measured before the applied pulsed electric fields and at the end of the recording, i.e., 5 min after the onset of the stimulus.

**Figure 6:**
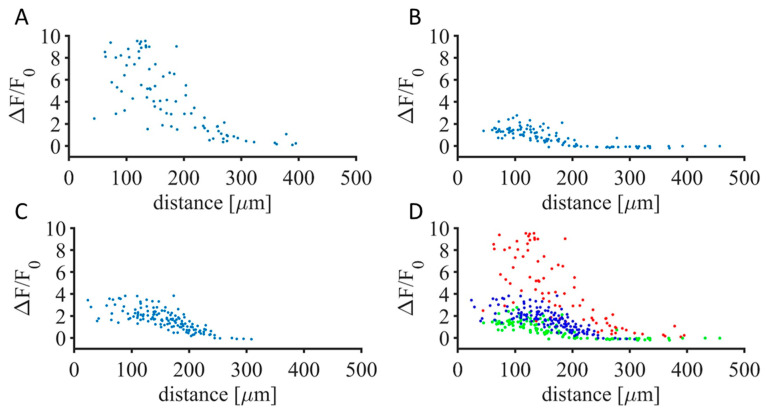
Evolution of GCaMP6f fluorescence intensity value 5 min after the stimulus onset in the neurons with regards to the distance from the active electrode. (**A**–**C**) represent each mouse dataset; (**D**) is the overlay of (**A**–**C**).

Figure 6, where each dot represents a single neuron, shows a maintained increase of GCaMP6f fluorescence intensity in time, dependent on the distance of the neurons from the active electrode. This increase, as measured 5 min after the H-FIRE onset, occurred in a radius of 200 µm from the active electrode, confirming that damage occurred in a highly focal way. Beyond 200 µm, the absence of GCaMP6f fluorescence increase confirmed the fact that the calcium transient returned to the baseline, and thus, sustained damage did not occur.

**Figure 7:**
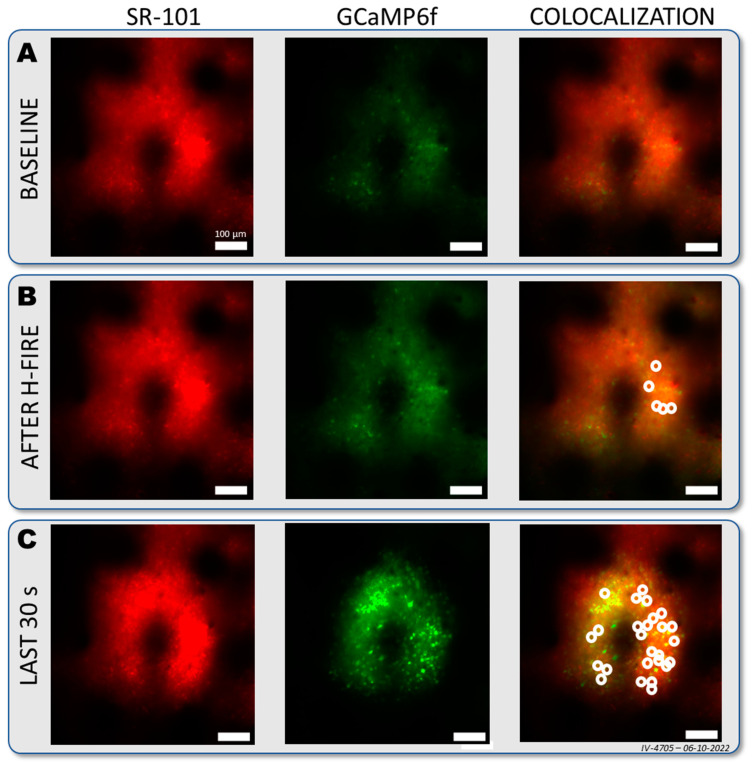
Double-labeled neurons appeared after H-FIRE. The figures show population tissue labeling with SR-101 (red) and the inherently expressed indicator GCaMP6f (green) and double-labeled tissue and cells (orange) before (**A**) and after (**B**) H-FIRE stimulation. (**C**) Tissue labeling 3 min 30 sec after the end of the H-FIRE stimulation protocol. White circles show doubl-labeled cells. Scale bars: 100 µm.

Using dual-channel two-photon imaging allowed the tracking of cell permeability through time. Since SR-101 is an astrocytic marker, there was no red labeling in neurons at the baseline (Figure 7A). The H-FIRE protocol immediately increased the red fluorescence in some neurons, showing that electropermeabilization occurred in vivo during the stimulation protocol. The number of neurons that took up the red staining increased substantially 3 min and 30 s after the end of the stimulation period. The local nature of the double-labeled cells suggested that permeabilization happened at the near vicinity of the stimulation site (Figure 7C), confirming the previous results from the observed increase in calcium signal (Figure 6).

**Figure 8:**
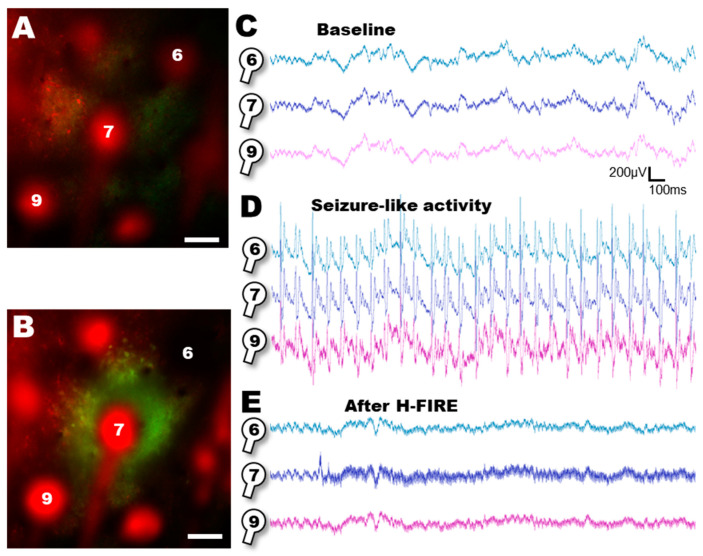
Recording of seizure-like activity. Two-photon recording of the neuronal tissue and electrode placement at baseline (**A**) and after the H-FIRE protocol (**B**). 6, 7 and 9 are electrode pad numbers. (**C**) Baseline LFP recording from the three recording sites labeled on (**A**). (**D**) Seizure-like activity recorded by the device. (**E**) LFP recording after the H-FIRE protocol evoked on electrode pad #7. Scale bar on (**A**) and (**B**): 100 µm.

The results above suggest that the H-FIRE protocol can evoke local calcium activity and does have electroporative effects in our experimental setting in vivo. Finally, we set out to explore the effects of H-FIRE on a well-described model of local 4AP injection that has been proven to evoke local seizure-like activity (SLA). After placing our device, we set out to record local field potentials from the two-photon imaging area (Figure 8A,C). Following the local 4-AP injection, we recorded LFP activity and found seizure-like activity (SLA) (Figure 8D), as expected [48]. Since our flexible electrode device was designed to be capable of both electrophysiological recording and electrical stimulation, we connected a channel to our stimulator and to the implanted frontal screw, while disconnecting it from the recording setup. This was necessary since the electrophysiological amplifier was not designed to withstand 150V inputs. We continued with the H-FIRE protocol, and captured two-photon images at the same time (Figure 8B). Finally, 30 min after the H-FIRE protocol, we reconnected the amplifier and continued LFP recordings on the site of the stimulation and around it (Figure 8E), observing that seizure-like activity was not apparent after the H-FIRE protocol.

## 4. Discussion

Our results demonstrate the in vivo application of a novel neurotechnological device with potential application in the treatment of epilepsy. Designed to be multi-channel, transparent and flexible, it offers several advantages worth exploring epilepsy and potentially other neurological disorders.

### 4.1. Targeted Intervention for Epilepsy

The proposed device possesses unique attributes tailored for epilepsy therapy. We designed a multichannel device to allow for multisite recording and multifocal stimulation. The pitch of 400 μm enables monitoring and intervention in specific brain regions associated with seizure initiation or propagation. The improved localization of epileptic networks by the mapping of multiunit activity in the extended region of the MEA allows for a local ablation protocol. This precise targeting potentially minimizes unintended effects compared to broader stimulation methods, as demonstrated in studies using deep brain stimulation (DBS) for epilepsy, where a targeted approach can lead to higher therapeutic effects and fewer side effects [59]. The end results are less vascular damage and direct ablation on the epileptogenic zone neurons only, which leads to less chance for local inflammation and improved recuperation and healing for the patients [30,40].

Our results showed that there was a long-lasting increase both in green and red fluorescence levels within a 200 µm range of the stimulation site. Regarding the green fluorescence increase, this was a clear sign that the GCaMP6f Ca^2+^ indicator was saturated by calcium, suggesting constantly elevated Ca^2+^ levels. In the literature, we can find multiple causes for increased Ca^2+^ levels. Firstly, they can arise from repeated firing of neurons, where the delay between action potentials is not long enough for the intracellular calcium levels to return to baseline levels. In this case, there is a gradual increase of green fluorescence with a tendency towards saturation—in other words, the fluorescence intensity shows a steplike increase, with each action potential corresponding to a calcium increase, but returns to the baseline after the last action potential [60] with the standard indicator decay constant. Secondly, the release from internal calcium stores can also be a source of green fluorescence in case of GCaMP-expressing neurons, but the exact observations require a specific, endoplasmic reticulum-targeted version of GCaMP [61]. Here, stimulation induces a calcium release from the internal calcium stores, but the calcium levels return to baseline levels within 10 s from the stimulation onset, which is far from our observations of multiple minutes. Finally, elevated Calcium levels can also be observed during induced cell death [62] or apoptosis [63]. In both cases, the baseline calcium levels rest elevated for the final minutes of the neuron till its degradation. Since we saw no decline in baseline fluorescence for minutes in the neurons within 200 µm of the H-FIRE-inducing contact site of the MEA, our results suggested that it was neuronal cell death that was responsible for the high green fluorescence observed.

Furthermore, we employed another dye (Sulforhodamine-101) that is reportedly an astrocytic marker and is not taken up by healthy neurons [47]. The red marker was clearly shown to be taken up by the same neurons that showed long-lasting elevation in green fluorescence, suggesting that their membrane became permeabilized. As H-FIRE is designed to irreversibly permeabilize cell membranes and we saw a marker—that under normal conditions cannot enter the cells—stain the neurons, we postulate that their membrane was permeabilized.

Our results suggest that the monitoring of the two fluorescence channels is adequate for determining that neurons are affected with terminal stimulation and degrade either with apoptosis or necrosis. Substantiated by previous data from the literature that irreversible electroporation causes cell death [26], we postulate that the H-FIRE protocol is capable of targeted ablation in a 200 µm area around the contact site.

Since the influence range of the microelectrodes deteriorates beyond 200 μm, this could avoid wide-scale and non-targeted ablation of neuronal tissue. At the same time, it allows for the targeting of specific neuronal populations within an epileptic focus, potentially suppressing aberrant activity while preserving healthy neighboring tissue. This approach aligns with the concept of network-targeted therapies, which aims to disrupt pathological networks within the brain while minimizing the disruption of healthy networks [64].

### 4.2. H-FIRE as a Means of Focal Ablation in Epilepsy

H-FIRE is a high-frequency variant of irreversible electroporation techniques that has shown particular utility in excitable tissues like the brain [31]. By delivering a rapid series of bipolar electrical pulses at repetition rates in the hundreds of kHz range, H-FIRE prevents muscle contractions and their associated pain when delivered to the sensorimotor cortex of rats. The high-frequency, repeated stimulation prevents repeated depolarization by disrupting the normal electrical activity of neurons. The electric field delivered by these pulses induces a transmembrane potential causing irreversible electroporation of the neuronal cell membranes. This leads to a loss of membrane integrity, the ionic gradients across the membrane, and cell death [26,27]. H-FIRE has previously been shown to be an effective means of non-thermally ablating brain tissue in the treatment of glioblastoma [28] and other cancers [37,40,65]. In our work, we show for the first time that H-FIRE is highly effective for the focal, non-thermal ablation of epileptic tissue when delivered by flexible implant with organic electrodes.

### 4.3. Effective and Verifiable Ablation

The ablation protocol used in our study—H-FIRE—offers several benefits over conventional electrical stimulation. As previously shown, H-FIRE induces permanent membrane permeabilization in targeted neurons, potentially enabling sustained inhibition of epileptic activity, compared to the transient effects of conventional approaches, as observed in studies using optogenetics for epilepsy treatment [66]. Moreover, the transparency of the substrate allows for real-time monitoring of stimulation effects using two-photon imaging techniques like those employed in the study [42]. This visual confirmation not only ensures proper device placement but also offers valuable insights into the spatiotemporal dynamics of neuronal response, aiding in optimizing stimulation parameters, similar to closed-loop DBS systems that use real-time feedback to adjust stimulation based on brain activity [67].

Electrical stimulation of the brain can lead to secondary effects, particularly temperature changes, which significantly impact neuronal activity. Previous studies using different electrode configurations with the same H-FIRE protocol show temperature increases up to 3 degrees Celsius [25], which cannot be responsible for neuronal death in itself [68]. Studies show that hyperthermia can increase neuronal excitability and can even aggravate febrile epileptic seizures [69], though others report that susceptibility to epileptogenesis is age-dependent and small levels of hyperthermia are countered by the increase in local inhibition [70]. At the same time, hyperthermia can cause the depletion of ATP, higher caspase-3 activation, and thus increased neuronal death by apoptosis [71]. In conclusion, slight temperature increase evoked by the H-FIRE protocol used in our study can modulate neuronal excitability, and it might be partially responsible for the observed signs for neuronal death.

### 4.4. Possible Impact on Other Neurological Diseases

The device presented in this work has been designed to be applicable for treating other neurological conditions. Similar targeted stimulation strategies could be used in chronic pain management, where specific neural pathways contribute to pain perception. For instance, studies have shown promising results using spinal cord stimulation (SCS) for chronic pain, with targeted SCS demonstrating improved efficacy and reduced side effects compared to traditional SCS [72]. Additionally, the ability to modulate specific neuronal populations could be explored in neurodegenerative disorders like Parkinson’s disease, where targeted stimulation of specific nuclei has proven to be an effective treatment method [73].

### 4.5. Scientific Considerations and Future Directions

Our flexible MEA is capable of both recording and stimulation; therefore, one could imagine that it could be used to localize epileptic foci prior to delivering the H-FIRE protocol for local epilepsy treatment for the cortical surface. This strategy could well be used for epileptogenic regions located in the temporal lobe near the cortical surface. Nevertheless, an inherent limitation to the use of surface MEAs arises when considering deeper brain regions such as the hippocampus. On the other hand, only two-thirds of the patients undergoing the resection of epileptic foci for medial temporal lobe epilepsy (medial TLE) become seizure-free in the first two to three years after surgery [74], and up to 40% of patients with TLE continue to experience persistent postoperative seizures at 2-year follow-up [75], with the size of resection being an important factor [76]. Here, flexible MEAs could serve as post-operational observation and treatment devices. In a future scenario, implanting flexible MEAs into the surgical area would allow the controlled electrophysiological monitoring of the local tissue. What is more, in case of recurring seizures, it would provide a means for the proper localization of new seizure foci and their immediate treatment by the H-FIRE ablation protocol. In conclusion, though deeper brain regions are not immediately accessible for our device, it could provide a solution for post-operative control and treatment.

Several considerations demand further research, such as the chronic implantation of the device, if necessary, to prove its feasibility in conditions such as Parkinson’s disease. At the same time, long-term effects of H-FIRE on neuronal health and tissue integrity require further experiments under in vivo conditions. Furthermore, translating findings from mice to humans necessitates scaling up the device and optimizing stimulation parameters for larger brains. Computational modeling and simulations can play a significant role in this process [26,33], as demonstrated in optimizing DBS parameters for human trials [77]. Additionally, integrating biocompatible feedback mechanisms would be crucial for closed-loop control and personalized treatment optimization, similar to advancements in closed-loop DBS systems [78].

## 5. Conclusions

Our flexible microelectrode array device was proven to be capable of both LFP recordings and local electroporation of nervous tissue in vivo. Its targeting capabilities hold significant potential for epilepsy treatment and potentially other neurological disorders. Future research addressing the mentioned considerations will pave the way for clinical translation and could potentially provide a means for the treatment of focal epilepsy.

## Data Availability

The raw data files for electrophysiological recordings and two-photon imaging and the custom codes and scripts are available on request.

## Supplementary Materials

The following supporting information can be downloaded at: https://www.mdpi.com/article/10.3390/s25010004/s1, Movie S1: H-FIRE-induced calcium response of neurons.
